
JOURNAL INFORMATION
==============================
NLM Title Abbreviation: J Diabetes Investig
Journal ID: J Diabetes Investig
NLM Journal ID: 101520702
Journal ID: JDI

Journal of Diabetes Investigation

ISSN: 2040-1116
EISSN: 2040-1124
Publisher: Wiley

ARTICLE INFORMATION
==============================
PMCID: PMC11474568
DOI: 10.1111/jdi.14141
Article ID: JDI14141
Article version: 1
Subjects: Issue Information, Issue Information

Issue Information

Electronic publication date: 2024 Jan 2
Publication date: 2024 Jan
Volume: 15
Issue: 1
Issue title: Diabetes and Big Data
First page: 1
Last page: 2
Copyright: © 2024 The Authors. Journal of Diabetes Investigation published by Asian Association for the Study of Diabetes (AASD) and John Wiley & Sons Australia, Ltd
License: This is an open access article under the terms of the http://creativecommons.org/licenses/by/4.0/ License, which permits use, distribution and reproduction in any medium, provided the original work is properly cited.
License URL: https://creativecommons.org/licenses/by/4.0/

Figure count: 0
Table count: 0
Page count: 5

source-schema-version-number: 2.0
cover-date: January 2024
details-of-publishers-convertor: Converter:WILEY_ML3GV2_TO_JATSPMC version:6.4.9 mode:remove_FC converted:15.10.2024

==============================

### JDI

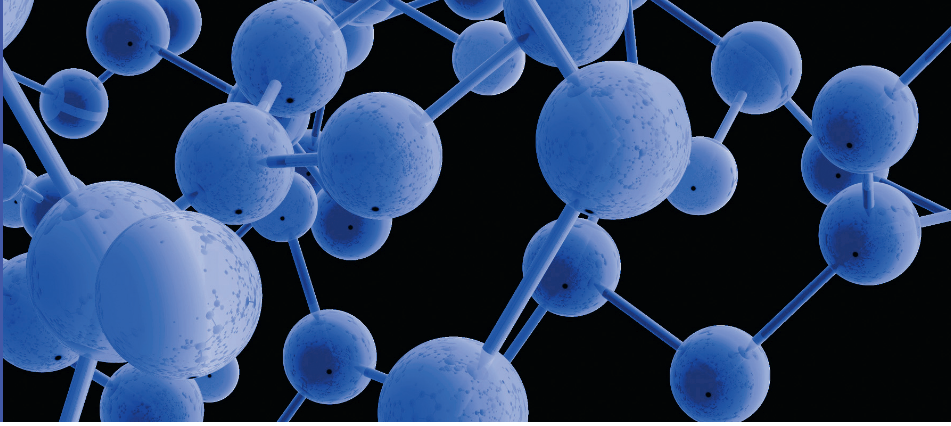

Volume 15 | Issue 1
January 2024

### Journal of Diabetes Investigation

Official Journal of the Asian Association for the Study of Diabetes

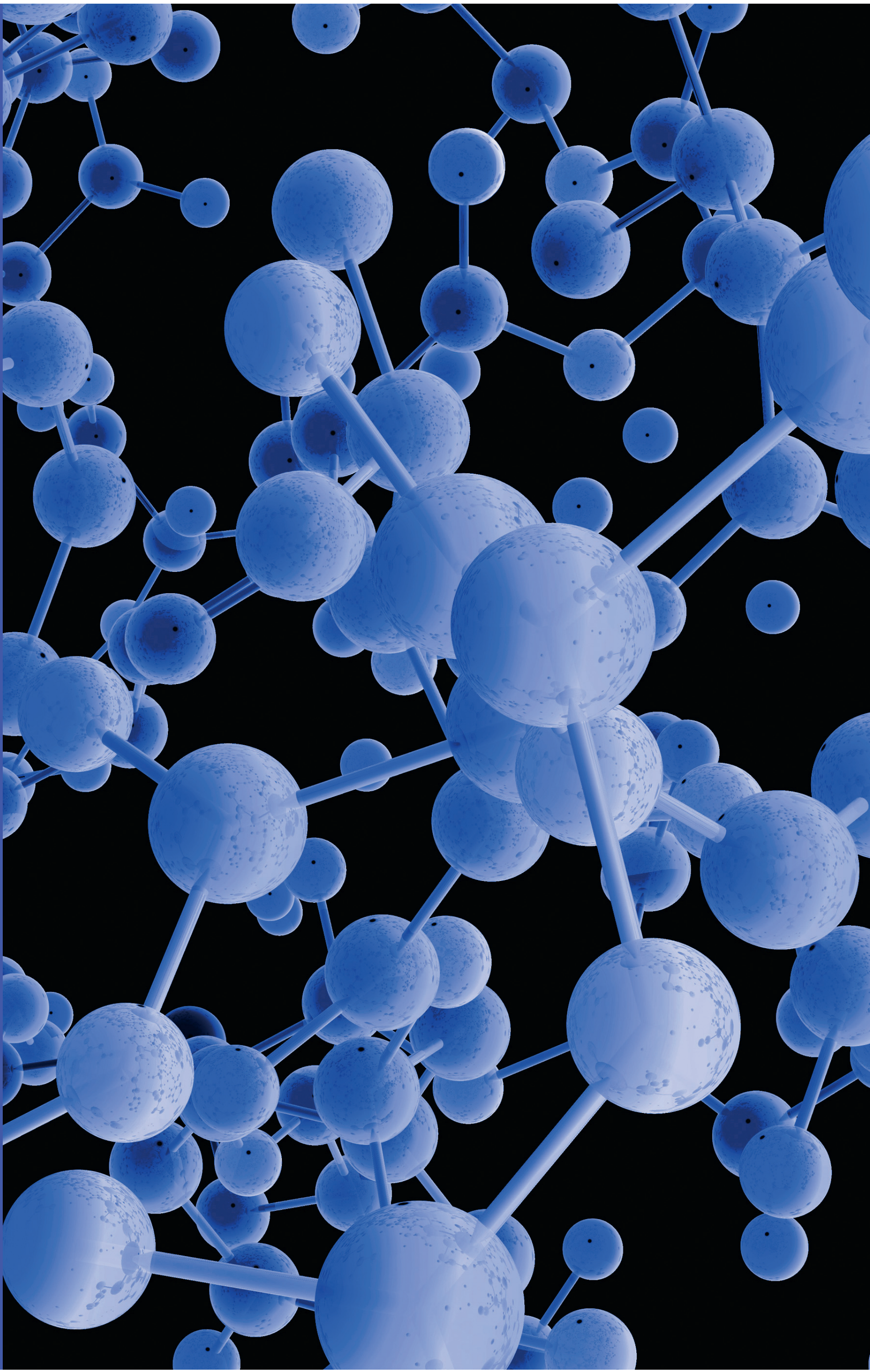

### Special Issue:
### Diabetes and Big Data

WILEY

**JDI Journal of Diabetes Investigation** Open access
**Official Journal of the Asian Association for the Study of Diabetes**

**Editor-in-Chief**
Nigishi Hotta, *Japan Organization of Occupational Health and Safety, Chubu Rosai Hospital, Nagoya, Japan*

**Co-Editor**
Domenico Accili, *Columbia University, New York, USA*

**Expert Editor**
Bo Ahren, *Lund, Sweden*
Young Min Cho, *Seoul, Korea*
Sean Dinneen, *Galway, Ireland*
Marc Gilbert, *Montpelier, France*
Limei Liu, *Shanghai, China*
Ronald Ma, *Hong Kong, China*
Louis Philipson, *Chicago, USA*
Wayne H-H Sheu, *Taichung, Taiwan*
Bernard Thorens, *Lausanne, Switzerland*
Daisuke Yabe, *Gifu, Japan*

**Editorial Board**
Eiichi Araki, *Kumamoto, Japan*
Xiaoping Chen, *Beijing, China*
Lee-Ming Chuang, *Taipei, Taiwan*
Masakazu Haneda, *Asahikawa, Japan*
Larry Low-Tone Ho, *Taipei, Taiwan*
Tianpei Hong, *Beijing, China*
Akihisa Imagawa, *Osaka, Japan*
Nobuya Inagaki, *Kyoto, Japan*
Linong Ji, *Beijing, China*
Weiping Jia, *Shanghai, China*
Yi-Der Jiang, *Taipei, Taiwan*
Takashi Kadowaki, *Tokyo, Japan*
Kohei Kaku, *Kurashiki, Japan*
Keizo Kanasaki, *Izumo, Japan*
Doo-Man Kim, *Seoul, Korea*
Karen Lam, *Hong Kong, China*
Hong Kyu Lee, *Gyeonggi-do, Korea*
In-Kyu Lee, *Daegu, Korea*
Moon-Kyu Lee, *Seoul, Korea*
Jiro Nakamura, *Nagakute, Japan*
Kishio Nanjo, *Wakayama, Japan*
Wataru Ogawa, *Kobe, Japan*
Yutaka Seino, *Osaka, Japan*
Shyi-Jang Shin, *Kaohsiung, Taiwan*
Hirohito Sone, *Niigata, Japan*
Kathryn Tan, *Hong Kong, China*
Yukio Tanizawa, *Ube, Japan*
Kohjiro Ueki, *Tokyo, Japan*
Jun Wada, *Okayama, Japan*
Hirotaka Watada, *Tokyo, Japan*
Jianping Weng, *Guangzhou, China*
Soroku Yagihashi, *Hirosaki, Japan*
Hiroshi Yamamoto, *Kanazawa, Japan*
Toshimasa Yamauchi, *Tokyo, Japan*
Wenying Yang, *Beijing, China*
Kazuki Yasuda, *Tokyo, Japan*
Koutaro Yokote, *Chiba, Japan*
Kun-Ho Yoon, *Seoul, Korea*
Dalong Zhu, *Nanjing, China*

**International Editorial Board**
Michael A. Brownlee, *New York, USA*
Subrata Chakrabarti, *Ontario, Canada*
Mark E. Cooper, *Prahran, Australia*
Philippe Froguel, *London, UK*
Wilfred Fujimoto, *Hawaii, USA*
Michael S. German, *San Francisco, USA*
Andrew Hattersley, *Exeter, UK*
Jean-Claude Henquin, *Brussels, Belgium*
Jens Juul Holst, *Copenhagen, Denmark*
Frank B. Hu, *Boston, USA*
C. Ronald Kahn, *Boston, USA*
George L. King, *Boston, USA*
Rayaz A. Malik, *Manchester, UK*
Michael Mauer, *Minneapolis, USA*
Carl Erik Mogensen, *Aarhus, Denmark*
Steven E. Shoelson, *Boston, USA*
Jay S. Skyler, *Miami, USA*
Paul Zimmet, *Melbourne, Australia*

**Assistant Editorial Board**
Tien-Jyun Chang, *Taipei, Taiwan*
Yoshiyuki Hamamoto, *Osaka, Japan*
Norio Harada, *Kyoto, Japan*
Yi-Jen Hung, *Taipei, Taiwan*
Chi-Min Hwu, *Taipei, Taiwan*
Hideki Kamiya, *Aichi, Japan*
Dae Jung Kim, *Suwon, Korea*
Seung-Hwan Lee, *Seoul, Korea*
Hung-Yuan Li, *Taipei, Taiwan*
Takashi Matsuzaka, *Tsukuba, Japan*
Keiko Naruse, *Aichi, Japan*
Takeshi Nishikawa, *Kumamoto, Japan*
Eung-Jung Rhee, *Seoul, Korea*
Yusuke Seino, *Aichi, Japan*
Hironori Waki, *Tokyo, Japan*

**Aims and Scope:** *Journal of Diabetes Investigation* is your core diabetes journal from Asia; the official journal of the Asian Association for the Study of Diabetes (AASD). The journal publishes original research, country reports, commentaries, reviews, mini-reviews, case reports, letters, as well as editorials and news. The journal embraces clinical and experimental research in diabetes and related areas. This includes aspects of prevention, treatment, as well as molecular aspects and pathophysiology. Translational research focused on the exchange of ideas between clinicians and researchers is also welcome.

**Address for Editorial Correspondence:**
The Editor, *Journal of Diabetes Investigation*, Nomura Fudosan Nishi Shinjuku Building, 8F 8-4-2 Nishi Shinjuku, Shinjuku-ku, Tokyo 160-0023, Japan. Tel: +81 3 4520 9009; Fax: +81 3 4520-9059 Email: JDI@wiley.com

**Copyright © 2024 Asian Association for the Study of Diabetes and John Wiley & Sons Australia, Ltd**

**Cover:** Insulin molecule © Pasieka/Science Photo Library

This journal is available online at Wiley Online Library. Visit wileyonlinelibrary.com to search the articles and register for table of contents and e-mail alerts.

**For submission instructions, and all other information visit wileyonlinelibrary.com/journal/jdi**

ISSN 2040-1124 (Online)

JDI.JEB.Jan24

WILEY

**Publisher**
*Journal of Diabetes Investigation* is published by John Wiley & Sons Australia, Ltd
Level 19, 15 William Street,
Melbourne, Victoria 3000,
Australia.
Tel: +61 (0)3 9274 3100

**Production Editor**
Jayalakshmi Mohan (email: JDIproduction@wiley.com)

**Abstracting and Indexing Services**
The Journal is indexed by Academic OneFile (GALE Cengage), Biological Abstracts (Clarivate Analytics), Biological Science Database (ProQuest), BIOSIS Previews (Clarivate Analytics), Embase (Elsevier), Health & Medical Collection (ProQuest), Health Research Premium Collection (ProQuest), Hospital Premium Collection (ProQuest), MEDLINE/PubMed (NLM), Natural Science Collection (ProQuest), ProQuest Central (ProQuest), Science Citation Index Expanded (Clarivate Analytics), SciTech Premium Collection (ProQuest), SCOPUS (Elsevier), Web of Science (Clarivate Analytics).

**Open Access and Copyright**
All articles published by *Journal of Diabetes Investigation* are fully open access: immediately freely available to read, download and share. All *Journal of Diabetes Investigation* articles accepted from 24 September 2013 are published under the terms of the Creative Commons Attribution 4.0 Unported (CC BY 4.0) license, which permits use, distribution and reproduction in any medium, provided the original work is properly cited. Articles accepted before this date were published under the agreement as stated in the final article. Copyright on any *Journal of Diabetes Investigation* article accepted after 24 September 2013, the date on which *Journal of Diabetes Investigation* became an open access journal, is retained by the author(s). Authors grant to *Journal of Diabetes Investigation* and Wiley a license to publish the article and for Wiley to identify itself as the original publisher. Authors also grant any third party the right to use the article freely as long as its integrity is maintained and its original authors, citation details and publisher are identified. Please visit https://authorservices.wiley.com/author-resources/Journal-Authors/open-access/hybrid-open-access.html for further information about Open Access.

**Use by commercial ''for-profit'' organisations (not applicable to CC BY 4.0 licensed articles)**
Use of Wiley Open Access articles for commercial, promotional, or marketing purposes requires further explicit permission from Wiley and will be subject to a fee. Requests to reproduce material from this journal content are being handled through the Rightslink® online service.

- Locate the article you wish to reproduce on Wiley Online Library (http://onlinelibrary.wiley.com)
- Navigate to the abstract page
- Click on the 'Request Permissions' link, under the 'ARTICLE TOOLS' menu
- Follow the online instructions and select your requirements from the drop down options and click on 'quick price' to get a quote
- Create a RightsLink® account to complete your transaction (and pay, where applicable)
- Read and accept our Terms & Conditions and Download your license
- For any technical queries please contact customercare@copyright.com
- For further information and to view a Rightslink® demo please visit www.wiley.com and select Rights & Permissions.
- Print reprints of Wiley Open Access articles can be purchased from corporatesales@wiley.com.

**Disclaimer**
The Publisher, the Asian Association for the Study of Diabetes, and Editors cannot be held responsible for any errors in or any consequences arising from the use of information contained in this journal. The views and opinions expressed do not necessarily reflect those of the Publisher, the Asian Association for the Study of Diabetes, Editors, or Authors neither does the publication of advertisements constitute any endorsement by the Publisher, the Asian Association for the Study of Diabetes, or Editors of the products advertised.

Wiley Open Access articles posted to repositories or websites are without warranty from Wiley of any kind, either express or implied, including, but not limited to, warranties of merchantability, fitness for a particular purpose, or noninfringement.
To the fullest extent permitted by law Wiley disclaims all liability for any loss or damage arising out of, or in connection, with the use of or inability to use the content.

**Journal Customer Services:** For ordering information, claims and any enquiry concerning your journal subscription please visit our Online Customer Help at https://wolsupport.wiley.com/s/contactsupport or contact your nearest office.
**Americas:** Email: cs-journals@wiley.com; Tel: +1 877 762 2974
**Europe, Middle East and Africa:** Email: cs-journals@wiley.com; Tel: +44 (0) 1243 684083.
**Germany, Austria, Switzerland, Luxembourg, Liechtenstein:** cs-germany@wiley.com; Tel: 0800 1800 536 (Germany)
**Asia Pacific:** Email: cs-journals@wiley.com; Tel: +65 3165 0890.
**Japan:** For Japanese speaking support, Email: cs-japan@wiley.com; .
**For a full list of frequently asked questions,** please visit https://wolsupport.wiley.com/s/

Wiley's Corporate Citizenship initiative seeks to address the environmental, social, economic, and ethical challenges faced in our business and which are important to our diverse stakeholder groups. Since launching the initiative, we have focused on sharing our content with those in need, enhancing community philanthropy, reducing our carbon impact, creating global guidelines and best practices for paper use, establishing a vendor code of ethics, and engaging our colleagues and other stakeholders in our efforts. Follow our progress at www.wiley.com/go/citizenship

VOLUME 15 – ISSUE 1 – JANUARY 2024

### Journal of Diabetes Investigation

Open access

### Special Issue: Diabetes and Big Data

Issue edited by: Professor Weiping Jia

### Reviews

**Glycemic variability: Measurement, target, impact on complications of diabetes and does it really matter?**
Yifei Mo, Jingyi Lu, and Jian Zhou 5

**The role of lipid dysregulation in gestational diabetes mellitus: Early prediction and postpartum prognosis**
Ziyi Zhang, Zheng Zhou, and Hong Li 15

### Original Articles

**Decline in measured glomerular filtration rate is associated with simple renal cyst in Chinese patients with type 2 diabetes**
Fu Qiaoling, Lu Jingyi, Li Yan, Li Wen, Yang Chaoying, Zhang Mingliang, and Zhang Lei 26

**Association of *RPS26* gene polymorphism with different types of diabetes in Chinese individuals**
Rong Song, Lingxiang Xie, Jin Ding, Yan Chen, Hailan Zou, Haipeng Pang, Yiman Peng, Ying Xia, Zhiguo Xie, Xia Li, Yang Xiao, Zhiguang Zhou, and Jingyi Hu 34

**Association of serum amyloid A and prognosis in people with diabetes and COVID-19: A retrospective cohort study**
Duoduo Qu, Jia Liu, Lihua Zhou, Yaling Yang, Chenwei Wu, Xinyue Xu, Qin Zhu, Chunhong Wang, and Xiaolong Zhao 44

**Identification of eight genomic protective alleles for mitochondrial diabetes by Kinship-graph convolutional network**
Jiahao Wang, Dandan Yan, Haoyue Cui, Rong Zhang, Xiaojing Ma, Luonan Chen, Cheng Hu, and Jiarui Wu 52

### REGULAR ARTICLES

### Commentaries

**Long-term safety and efficacy of SGLT2 inhibitor use in older east Asians with type 2 diabetes**
Yoshihiro Takahashi, Yutaka Seino, and Daisuke Yabe 63

**Pituitary gland agouti-related peptide cells: Novel player controlling glucose metabolism**
Kei Takahashi and Hideki Katagiri 67

### Original Articles

#### Basic Science and Research

**Exploration of the mechanism of reinfusion of fresh autologous blood in type 2 diabetes mice to induce macrophage polarization and inhibit erythrocyte damage**
She-jun Hu, Jia-ming Xu, Man-di Wu, Ke Yue, Ying-hui Cui, Yu Bai, Lai-wei You, and Jian-Rong Guo 70

#### Clinical Science and Care

**High sensitivity C-reactive protein and prediabetes progression and regression in middle-aged and older adults: A prospective cohort study**
Zi-Jian Cheng, Yan-Fei Wang, Xi-Yuan Jiang, Wen-Yan Ren, Shu-Feng Lei, Fei-Yan Deng, and Long-Fei Wu 78

**Identification and validation of voltage-dependent anion channel 1-related genes and immune cell infiltration in diabetic nephropathy**
Jiaqun Lin, Mengjie Weng, Jing Zheng, Kun Nie, Siyi Rao, Yongjie Zhuo, and Jianxin Wan **87**

**Sympathetic skin response for early detection of type 2 diabetic peripheral neuropathy and nephropathy**
Hongying Liu, Sheng Tan, Zhenyu Ma, Qingchun Gao, and Weihong Yang **106**

**Association between serum uric acid-to-high-density lipoprotein cholesterol ratio and insulin resistance in patients with type 2 diabetes mellitus**
Xinhe Zhou and Jing Xu **113**

**Hepatocyte nuclear factor 1B deletion, but not intragenic mutation, might be more susceptible to hypomagnesemia**
Yanfei Wang, Xiaoyu Xiao, Qiuqiu Lin, Rong Song, Xiaozhou Wang, Yiji Liang, Jingsong Chen, Xiaojun Luan, Zhiguang Zhou, Yang Xiao, Yaoming Xue, and Jingyi Hu **121**

**Type 1 diabetes, COVID-19 vaccines and short-term safety: Subgroup analysis from the global COVAD study**
Tulika Chatterjee, Naveen Ravichandran, Narmadha Nair, Abraham Edgar Gracia-Ramos, Bhupen Barman, Parikshit Sen, Mrudula Joshi, Sreoshy Saha, Arvind Nune, Arun Kumar R Pande, Tsvetelina Velikova, Ioannis Parodis, Ai Lyn Tan, Samuel Katsuyuki Shinjo, Hiya Boro, Vikas Agarwal, Rohit Aggarwal, Latika Gupta and COVAD Study Group **131**
